# Exosomes as Molecular Mediators of Reproductive Health: From Infertility to HPV-Related Disease

**DOI:** 10.3390/cimb48101031

**Published:** 2026-10-04

**Authors:** Serafoula-Natalia Siourtou, Sofoklis Stavros, Maria-Anastasia Daskalaki, Eriketi Kokkosi, Athanasios Zikopoulos, Despoina Mavrogianni, Vasileia Kalogerakou, Theodoros Karampitsakos, Efthalia Moustakli, Nefeli Arkouli, Anastasios Potiris, Georgios Daskalakis, Peter Drakakis

**Affiliations:** 1 Medical School, National and Kapodistrian University of Athens, 11528 Athens, Greece; serasiou@med.uoa.gr (S.-N.S.); vasileiakal@med.uoa.gr (V.K.); 2 Third Department of Obstetrics and Gynecology, University General Hospital “ATTIKON”, Medical School, National and Kapodistrian University of Athens, 12462 Athens, Greece; theokarampitsakos@hotmail.com (T.K.); apotiris@med.uoa.gr (A.P.); pdrakakis@hotmail.com (P.D.); 3 First Department of Obstetrics and Gynecology, Alexandra Hospital, Medical School, National and Kapodistrian University of Athens, 11528 Athens, Greece; anastasia.daskalaki00@gmail.com (M.-A.D.); dmavrogianni@med.uoa.gr (D.M.); gdaskalakis@yahoo.com (G.D.); 4 Department of Midwifery, Faculty of Health and Caring Sciences, University of West Attica, 12243 Athens, Greece; ekokkosi@uniwa.gr; 5 Torbay and South Devon NHS Foundation Trust Lowes Brg, Torquay TQ2 7AA, UK; thanzik92@gmail.com; 6 Department of Nursing, School of Health Sciences, University of Ioannina, 45500 Ioannina, Greece; ef.moustakli@uoi.gr; 7 Department of Obstetrics and Gynecology, Tzaneio General Hospital, 18536 Piraeus, Greece; narkoyli@windowslive.com

**Keywords:** exosomes, extracellular vesicles, human papillomavirus (HPV), reproductive medicine, precision medicine

## Abstract

This review provides a comprehensive overview of the biological roles of exosomes in reproductive physiology, examines their involvement in mechanisms associated with female and male infertility, and discusses their emerging role in human papillomavirus (HPV)-related reproductive disease. Exosomes are small extracellular vesicles that mediate intercellular communication by transporting proteins, lipids, DNA, messenger RNAs (mRNAs), microRNAs (miRNAs), long non-coding RNAs (lncRNAs), circular RNAs (circRNAs), and other bioactive molecules, thereby regulating the biological functions of recipient cells. Particular emphasis is placed on the roles of exosomes in folliculogenesis, gametogenesis, fertilization, embryo implantation, and spermatogenesis. Alterations in exosomal cargo and function have been reported in several female and male reproductive disorders. In parallel, emerging evidence suggests that exosomes may participate in HPV-associated disease through mechanisms involving viral persistence, modulation of immune and inflammatory responses, and transfer of viral and host-derived molecules between cells. Exosomes are also being investigated as potential non-invasive biomarkers and therapeutic delivery platforms; however, these applications remain largely investigational. Importantly, substantial heterogeneity in extracellular vesicle isolation, purification, and characterization methods limits cross-study comparability and remains a major barrier to clinical translation. Overall, current evidence supports exosome-mediated communication as a biologically relevant mechanism across reproductive physiology, infertility-related disorders, and HPV-associated disease, while further standardized and clinically validated studies are required to establish their diagnostic and therapeutic utility.

## 1. Introduction

Infertility affects approximately one in six individuals worldwide and represents a growing public health concern with profound medical, psychological, and socioeconomic consequences [1,2]. The maintenance of reproductive health depends on tightly regulated molecular communication between germ cells, somatic cells, immune cells, and the surrounding reproductive microenvironment. Disruption of these signaling networks contributes not only to female and male infertility but also to chronic inflammatory conditions, impaired implantation, reproductive tract malignancies, and persistent viral infections of the reproductive tract, particularly those caused by human papillomavirus (HPV) [1,3,4].

Among the mechanisms responsible for intercellular communication, extracellular vesicles (EVs) have emerged as fundamental regulators of reproductive physiology [5,6]. Exosomes are a subtype of small EVs of endosomal origin, released following the fusion of multivesicular bodies with the plasma membrane [5,6,7]. Their diverse molecular cargo, including proteins, lipids, messenger RNAs (mRNAs), microRNAs (miRNAs), long non-coding RNAs (lncRNAs), circular RNAs (circRNAs), DNA fragments, metabolites, and other bioactive molecules, enables the coordinated regulation of gene expression and cellular function in recipient cells [5,6,8,9]. However, the terminology used across the literature is not uniform, as many studies reporting “exosomes” rely on isolation methods that may yield heterogeneous EV populations containing other vesicle subtypes. This methodological heterogeneity should be considered when interpreting and comparing findings across studies. Consequently, exosomes have become recognized as important mediators implicated in physiological homeostasis, intercellular communication, and disease pathogenesis [5,10,11].

Within the reproductive system, exosome-mediated communication regulates numerous biological events, including folliculogenesis, oocyte maturation, sperm maturation, fertilization, embryo development, implantation, and maternal–fetal interactions [2,12,13,14,15]. Alterations in exosomal cargo and intercellular signaling have been associated with several reproductive disorders, including polycystic ovary syndrome, endometriosis, premature ovarian insufficiency, varicocele, azoospermia, and other causes of female and male infertility [16]. Beyond reproductive dysfunction, increasing evidence indicates that exosomes actively participate in host–pathogen interactions by mediating immune modulation, chronic inflammation, viral persistence, and the dissemination of regulatory molecules between infected and non-infected cells [17,18,19].

Persistent infection with HPV is the principal etiological factor underlying cervical cancer and has also been investigated in relation to adverse reproductive outcomes and infertility. However, the epidemiological evidence linking HPV infection directly to infertility remains heterogeneous, and a causal relationship has not been established. Recent studies have demonstrated that HPV-infected epithelial cells secrete exosomes containing viral nucleic acids, oncogenic transcripts, viral proteins, and regulatory non-coding RNAs that may influence the local microenvironment and PV-associated disease progression [20,21]. These findings have prompted increasing investigation of exosome-mediated signaling in HPV-associated disease and its potential relevance to reproductive health.

Although exosomes have been extensively investigated in reproductive disorders and viral infections separately, their roles across these conditions are generally discussed within distinct fields [3]. Infertility and HPV infection are clinically distinct entities; however, they share several exosome-mediated biological processes relevant to reproductive health, including altered intercellular communication, inflammatory and immune regulation, oxidative stress, and the transfer of regulatory non-coding RNAs. HPV therefore provides a clinically relevant viral model through which the role of exosome-mediated signaling in reproductive-tract infection and disease can be examined alongside its established and emerging roles in reproductive physiology and infertility. Other genital infections may also involve extracellular-vesicle-mediated mechanisms; however, a comprehensive analysis of these infections is beyond the scope of the present review [22,23].

Accordingly, this review does not aim to imply a direct equivalence between infertility and HPV infection, but rather examines them within a common exosome-centered framework. We provide a comprehensive overview of exosome biology and their roles in female and male reproductive physiology, infertility, and HPV-associated disease, followed by an evaluation of their emerging translational potential as diagnostic biomarkers and therapeutic platforms in reproductive medicine.

## 2. Biology of Exosomes

### 2.1. Biogenesis and Molecular Composition

Exosomes represent a unique subset of EVs and serve as key mediators of cell–cell communication through the transportation of biologically functional molecules from one cell to another. Exosomes are membrane-bound nanovesicles with a size of about 30–200 nm that have the same membrane structure as the donor cells because of their endosomal nature [5].

Exosome biogenesis begins with endocytosis and the formation of early endosomes, which subsequently mature into late endosomes or multivesicular bodies (MVBs) [24]. During this process, inward budding of the endosomal membrane generates intraluminal vesicles (ILVs), which selectively incorporate proteins, lipids, nucleic acids, and other bioactive molecules. Mature MVBs subsequently follow one of two distinct pathways: they either fuse with lysosomes for degradation or fuse with the plasma membrane, releasing ILVs into the extracellular space as exosomes [24,25].

The exosome consists of selective molecular cargo rather than the random sampling of the parent cell’s composition. This molecular cargo includes proteins, lipids, mRNA, miRNA, lncRNA, circRNA, DNA fragments, glycoconjugates, and other molecules that influence gene expression and cellular behavior in target cells [26,27,28]. This selective transport of biomolecules enables exosomes to function as efficient biological carriers, facilitating the transfer of molecular information between neighboring and distant cells.

### 2.2. Exosome-Mediated Intercellular Communication

After their secretion into the extracellular milieu, exosomes engage in crosstalk with the target cells through specific binding, membrane fusion, or endocytosis, thus providing the delivery of their cargo and controlling the activity of several signaling pathways inside the cells [29,30,31,32]. Through these mechanisms, exosomes regulate numerous biological processes, including cell proliferation, differentiation, apoptosis, immune regulation, tissue remodeling, and cellular homeostasis [33,34].

Within the reproductive system, exosomes orchestrate molecular communication among germ cells, somatic and immune cell populations, and surrounding reproductive tissues, thereby regulating the interactions required for normal reproductive function. By transferring regulatory RNAs, proteins, and other signaling molecules, exosomes play a central role in regulating gene expression and cellular responses in recipient cells [22,35,36].

Modifications in the components of these molecules will consequently interfere with cell–cell signaling processes and contribute to reproductive dysfunctions.

Growing evidence indicates that exosomes participate in host–pathogen interactions by modulating immune and inflammatory responses and facilitating the transfer of viral and host-derived molecules between infected and uninfected cells [2,37]. These properties have generated considerable interest in the role of exosomes in chronic viral infections, particularly HPV, where they contribute to viral persistence, immune evasion, and disease progression [37].

### 2.3. Biological and Clinical Significance of Exosomes

However, the biological functions of exosomes extend beyond physiological intercellular communication. Exosomes have been found to play important roles in developmental biology, immunology, tissue healing, and homeostasis, while abnormal regulation of exosome secretion and loading has been associated with various pathologies, including inflammation, neurodegeneration, and cancer [29,38,39].

**Figure 1:**
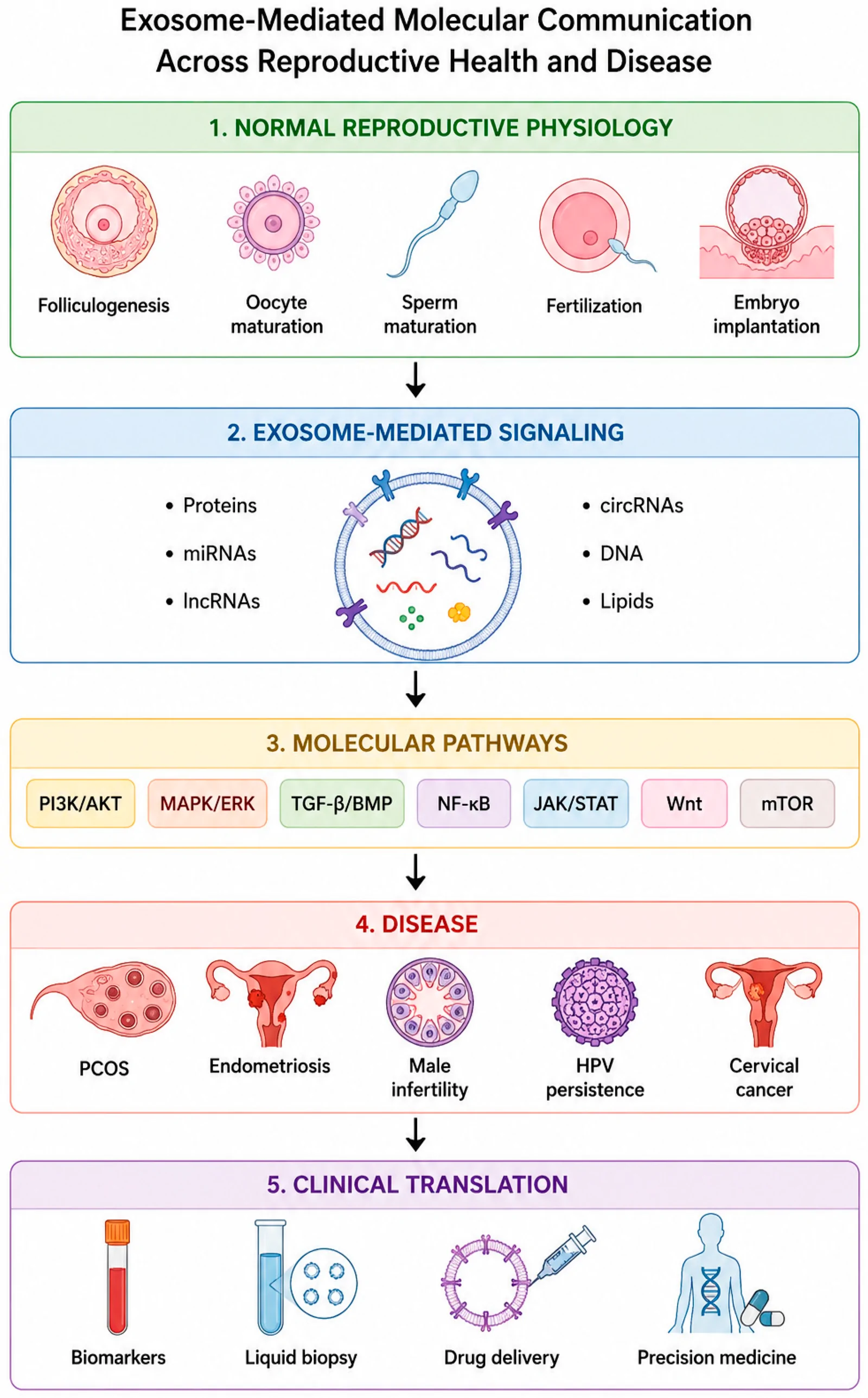
Exosome-mediated molecular communication across reproductive health and disease.

The unique properties of exosomes, including their ability to transport and protect bioactive molecular cargo, have generated considerable interest in their potential clinical applications. Although exosomes are often described as having favorable biocompatibility and immunogenicity profiles, these properties are context-dependent and may vary according to their cellular source, molecular cargo, recipient, and route of administration. Furthermore, exosome uptake is not inherently tissue-specific, and achieving reliable tissue- or cell-specific targeting remains an important challenge for therapeutic development. The molecular cargo of exosomes reflects the physiological and pathological status of their parent cells, making them promising non-invasive biomarkers for disease diagnosis, prognosis, and treatment monitoring [35]. Moreover, the ability of exosomes to transport nucleic acids, proteins, and therapeutic agents has prompted their investigation as delivery platforms; however, effective and specific targeting remains a major challenge for their therapeutic translation. Exosomes are therefore being investigated within the broader context of precision medicine, molecular diagnostics, and regenerative medicine [40,41]. Figure 1 illustrates the central role of exosomes as molecular mediators linking normal reproductive physiology, infertility, HPV-related reproductive diseases, and emerging precision diagnostic and therapeutic strategies.

## 3. Exosomes in Reproductive Physiology

### 3.1. Exosomes in Female Reproductive Physiology

Exosomes are increasingly recognized as mediators of intercellular communication within the female reproductive system, where they have been implicated in folliculogenesis, oocyte maturation, fertilization, embryo development, and endometrial receptivity [23,35,36]. Exosomes may contribute to bidirectional communication between oocytes, granulosa cells, cumulus cells, and other components of the follicular microenvironment by exchanging proteins, lipids, and nucleic acids [42,43].

Studies of extracellular vesicles derived from granulosa cells and follicular fluid have identified miRNAs associated with pathways involved in follicular development, including PI3K/AKT, MAPK/ERK, TGF-β/BMP, glycolysis, apoptosis, and cell-cycle regulation [13,44]. However, the supporting evidence is heterogeneous and includes human follicular-fluid studies as well as in vitro and animal models; consequently, mechanistic findings from experimental systems cannot necessarily be directly extrapolated to human ovarian physiology. The PI3K/AKT signaling pathway promotes granulosa cell proliferation and survival, enhances follicle-stimulating hormone (FSH) responsiveness, and supports steroidogenesis. Similarly, the MAPK/ERK signaling pathway mediates luteinizing hormone (LH)-induced follicular maturation, whereas the TGF-β/BMP pathway, primarily regulated by growth differentiation factor 9 (GDF9) and bone morphogenetic protein 15 (BMP15), coordinates communication between oocytes and surrounding somatic cells during follicular development [13,44]. Importantly, the identification or enrichment of particular miRNAs within exosome-enriched preparations does not by itself demonstrate that these molecules are delivered to recipient cells at concentrations sufficient to produce biologically relevant effects in vivo.

Experimental studies further suggest that exosome-mediated signaling may influence metabolic homeostasis within the ovarian follicle through the transfer of molecules associated with glycolytic metabolism and cellular survival pathways. Exosomes have also been implicated in apoptosis and cell-cycle regulation, processes relevant to granulosa cell viability, follicular atresia, and oocyte developmental competence [13,23,45]. Nevertheless, interpretation of these findings is complicated by substantial methodological heterogeneity. Follicular fluid contains diverse extracellular particles, including multiple EV subtypes and lipoproteins, which may be co-isolated depending on the separation method used. Differences in sample handling, isolation and purification protocols, EV characterization, and biological source also contribute to inter-study and batch-to-batch variability. Consequently, findings attributed specifically to exosomes should be interpreted cautiously when vesicle identity and purity have not been rigorously established.

### 3.2. Exosomes in Male Reproductive Physiology

In the male reproductive system, exosomes are primarily secreted by the epididymis, prostate, and seminal vesicles, where they contribute to sperm maturation, functional competence, and fertilization [2]. Epididymal exosomes, commonly referred to as epididymosomes, deliver proteins, lipids, messenger RNAs, and regulatory RNAs to developing spermatozoa during their transit through the epididymis, thereby promoting the acquisition of motility, membrane remodeling, and fertilizing capacity [2,14,46,47].

Because mature spermatozoa contain minimal cytoplasm and exhibit limited transcriptional and translational activity, exosome-mediated molecular transfer has been proposed as one mechanism through which spermatozoa may acquire additional molecular components during post-testicular maturation [48,49]. Epididymosomes have been reported to transfer structural proteins, enzymes, lipids, and non-coding RNAs to spermatozoa, potentially contributing to post-testicular modifications of sperm function [50].

Exosomes also participate in the selective quality control of spermatozoa. Through mechanisms involving the ubiquitin–proteasome pathway, they contribute to the identification and elimination of defective germ cells while simultaneously supporting membrane remodeling, capacitation, and preparation for the acrosome reaction required for successful fertilization [14,51].

Seminal plasma also contains prostasomes, EVs predominantly associated with prostatic secretion, which have been implicated in sperm membrane stabilization, protection against oxidative stress (OS), modulation of immune responses, and enhancement of sperm motility [2,51,52]. These vesicles are enriched in structural proteins, annexins, and transport proteins that facilitate interactions with spermatozoa and regulate the membrane remodeling required for fertilization [51,52]. Although epididymosomes and prostasomes are commonly distinguished according to their anatomical origin and proposed biological functions, their overlapping size and biophysical characteristics make their complete separation and independent characterization challenging in seminal samples. Consequently, studies of seminal EV preparations may contain heterogeneous vesicle populations, and functional effects cannot always be unequivocally attributed to a single EV subtype. In addition to their physiological functions, seminal exosomes contribute to the transmission of paternal environmental and age-related influences by altering the molecular cargo transferred to spermatozoa, thereby affecting sperm quality and reproductive outcomes [53,54].

Collectively, exosomes establish a complex communication network within the female and male reproductive systems by coordinating the molecular events required for gametogenesis, fertilization, embryo development, and reproductive homeostasis. Alterations in these signaling pathways have been associated with many reproductive disorders [22]. The principal physiological functions of exosomes within the female and male reproductive systems, together with their major molecular cargo and associated signaling pathways, are summarized in Table 1.

## 4. Exosomes in Female Reproductive Disorders

Exosome-mediated intercellular communication is essential for maintaining ovarian homeostasis and normal reproductive function. Alterations in exosome secretion, molecular cargo, or associated signaling pathways have been reported in several female reproductive disorders. Changes in the expression of exosomal proteins, messenger RNAs, and non-coding RNAs have been associated with processes involving cellular proliferation, apoptosis, inflammation, angiogenesis, steroidogenesis, and immune regulation, which may be relevant to impaired follicular development, reduced oocyte competence, defective implantation, and infertility [23,35,55].

### 4.1. Polycystic Ovary Syndrome

Polycystic ovary syndrome (PCOS) is one of the most common endocrine disorders affecting women of reproductive age and is characterized by chronic anovulation, hyperandrogenism, and polycystic ovarian morphology [56]. Growing evidence indicates that exosome-mediated signaling may be involved in PCOS through the transfer of regulatory RNAs associated with ovarian metabolism, steroidogenesis, inflammatory responses, and granulosa cell function [57,58,59]. However, most available evidence linking altered exosomal miRNA profiles to PCOS is associative or derived from in vitro experimental studies, and a causal role in the development of the syndrome has not been established.

Exosomes isolated from follicular fluid (FF) and adipose tissue have been reported to exhibit altered microRNA profiles associated with insulin sensitivity, apoptosis, and the expression of steroidogenic enzymes in granulosa cells, suggesting potential links between metabolic dysfunction and impaired folliculogenesis [16,58]. Additionally, exosomes transport lncRNAs and circRNAs associated with pathways involved in gene expression, DNA metabolism, and cell proliferation, supporting further investigation of their potential involvement in ovarian physiology and PCOS [3,60].

Among the molecular mechanisms investigated in PCOS pathogenesis, exosomal miR-143-3p has attracted considerable attention. Elevated expression of exosomal miR-143-3p has been reported in PCOS-associated FF, while experimental studies indicate that miR-143-3p can promote granulosa cell apoptosis and inhibit granulosa cell proliferation by targeting BMPR1A, thereby suppressing the Smad1/5/8 signaling pathway [61,62,63]. Although these findings provide mechanistic evidence at the cellular level, they do not establish that altered exosomal miR-143-3p is sufficient to induce the PCOS phenotype in vivo.

Experimental studies have also investigated engineered exosomes as potential therapeutic approaches. The use of exosomes loaded with microRNAs to regulate PI3K/AKT and ERK1/2 pathways has been explored for its potential to modulate granulosa cell viability, oocyte maturation, and follicular development in experimental settings [13]. Similarly, combining exosome-based therapy with photobiomodulation has demonstrated effects on OS, intracellular glutathione levels, and the expression of inflammation-related microRNAs implicated in ovarian dysfunction in experimental models [64]. These approaches remain at an early preclinical stage, and their safety, efficacy, reproducibility, and clinical applicability have not been established.

An additional challenge for exosome-based biomarker development is the substantial clinical and biological heterogeneity of PCOS. Different PCOS phenotypes may vary in their reproductive, endocrine, and metabolic characteristics, potentially resulting in distinct exosomal molecular profiles [55,56,58]. Consequently, a single exosomal biomarker may not adequately capture the full biological heterogeneity of the syndrome, and future studies should consider phenotype-specific molecular signatures and validation in well-characterized patient populations.

### 4.2. Endometriosis

Endometriosis is an inflammatory disease defined as the presence of endometrial-like tissue outside the uterine cavity and is one of the major causes of chronic pelvic pain and infertility in women of reproductive age [65]. The pathogenesis of endometriosis involves complex crosstalk among inflammatory, angiogenic, immunomodulatory, and cell signaling pathways, with exosome-mediated communication representing one of several mechanisms potentially involved in this multifactorial process [66,67].

Exosomes and other EVs derived from endometrial cells, macrophages, erythrocytes, and peritoneal mesothelial cells have been reported to transfer microRNAs, proteins, and lipids associated with modulation of the PI3K/AKT/mTOR, TGF-β/SMAD, MAPK/ERK, NOTCH, and NF-κB/JAK/STAT signaling pathways [32]. Experimental studies suggest that exosome-mediated signaling may contribute to processes such as fibroblast activation, macrophage polarization, epithelial–mesenchymal transition, angiogenesis, OS, progesterone resistance, and ectopic lesion survival [30,67,68]. However, these processes are also regulated by direct cell–cell interactions, cytokines, hormones, growth factors, and other soluble mediators within the peritoneal microenvironment; therefore, the relative contribution of exosomes to endometriosis pathophysiology remains incompletely defined.

Another potential mechanism involving exosome-mediated signaling relates to immune regulation in endometriosis. Experimental evidence indicates that exosomes derived from endometriotic lesions may contain NKG2D receptor ligands, including MICA/B and ULBP proteins, which can promote NKG2D receptor internalization and reduce natural killer (NK) cell cytotoxicity. Concurrently, exosomal Fas ligand (FasL) and TNF-related apoptosis-inducing ligand (TRAIL) have been reported to promote apoptosis in activated immune cells, suggesting a potential contribution to the immunoregulatory microenvironment associated with ectopic endometrial tissue [69].

Exosomes may also reflect molecular alterations within the endometrial and peritoneal microenvironments. Exosomal microRNAs detected in plasma, FF, and endometrial fluid have been associated with blastocyst quality, implantation rates, the number of metaphase II oocytes, and live birth outcomes [30]. However, interpretation and comparison of exosomal profiles across studies are limited by differences in EV isolation, purification, and characterization methods. In addition, endometriosis is clinically and biologically heterogeneous, with variation in disease phenotype, lesion location, stage, and patient characteristics, which may contribute to interindividual differences in EV cargo [70]. These factors currently limit the identification of reproducible endometriosis-specific exosomal signatures and their translation into clinically useful biomarkers.

### 4.3. Diminished Ovarian Reserve and Endometrial Receptivity

Recent studies have shown that alterations in exosome-mediated signaling are associated with diminished ovarian reserve (DOR), impaired oocyte competence, and implantation failure [71,72]. MicroRNA content in exosomes isolated from FF is associated with ovarian reserve and the ability to develop [71].

Recent studies have shown that exosomal microRNAs regulate the PI3K/AKT signaling pathway by inhibiting PTEN, thereby promoting granulosa cell survival and the generation of developmentally competent metaphase II oocytes [72]. Activation of the PI3K/AKT signaling pathway promotes cell proliferation and inhibits apoptosis, thereby contributing to oocyte maturation and developmental competence [72]. The principal exosome-mediated molecular mechanisms underlying female reproductive disorders, together with their biological functions and potential clinical applications, are summarized in Table 2.

Collectively, these findings indicate that exosome-mediated molecular interactions play central roles in both normal female reproductive physiology and the pathogenesis of reproductive disorders.

## 5. Exosomes in Male Reproductive Disorders

Exosomes are involved in several aspects of male reproductive function, contributing to sperm maturation, membrane remodeling, capacitation, fertilization, and protection against oxidative and immunological stress. Consequently, alterations in exosome biogenesis or molecular cargo have been associated with impaired sperm quality and several causes of male infertility. Altered exosomal profiles have been associated with changes in sperm motility, morphology, DNA integrity, OS responses, and post-transcriptional gene regulation, suggesting their potential relevance to male reproductive potential [2,35,73].

### 5.1. Azoospermia and Sperm Dysfunction

Azoospermia represents one of the most severe forms of male infertility and requires accurate differentiation between obstructive and non-obstructive etiologies. Although current evaluation relies on clinical assessment, endocrine and genetic testing, and imaging where indicated, these approaches may not fully characterize the underlying spermatogenic status or reliably predict the presence of sperm within testicular tissue. In this context, seminal plasma exosomes have emerged as potential non-invasive sources of molecular information because they reflect the physiological status of the male reproductive tract and carry proteins and regulatory RNAs associated with reproductive function and disease [51,74,75]. However, seminal plasma contains EVs originating from multiple tissues and accessory glands of the male reproductive tract, which complicates attribution of individual exosomal signatures specifically to testicular or spermatogenic dysfunction. This biological heterogeneity represents an important limitation when evaluating seminal EVs as biomarkers of azoospermia.

Several studies have shown that distinct exosomal miRNA expression profiles are associated with different etiologies of azoospermia, suggesting their potential value for investigating the molecular mechanisms underlying spermatogenesis and testicular function [76,77]. Moreover, the levels of expression of CRISP (cysteine-rich secretory protein), which is needed for sperm motility and fertilization, are significantly higher in exosomes taken from the seminal plasma of fertile men. Conversely, glycodelin has been predominantly identified in exosomes from men with severe asthenozoospermia, suggesting that alterations in exosomal protein cargo may be associated with underlying sperm dysfunction [78,79,80].

Exosomal cargo has also been associated with signaling pathways involved in spermatogenesis, including Wnt, MAPK, and mTOR signaling [2,35]. Alterations in exosomal miRNA expression may influence components of these signaling networks; however, these pathways are regulated by numerous cellular and molecular mechanisms, and their involvement does not demonstrate exosome-specific regulation or causality. Therefore, the specific contribution of exosome-mediated signaling to sperm production, motility, and fertilization potential remains to be established.

### 5.2. Varicocele and OS

Varicocele is one of the leading causes of male infertility due to progressive OS, altered spermatogenesis, and sperm DNA damage. Limited evidence suggests that varicocele may also be associated with alterations in the molecular profile of seminal plasma EVs; however, data specifically addressing exosome involvement remain scarce [73,81].

In one study, alterations in several exosome-associated proteins, including kinesin family member 5B (KIF5B), annexin A2 (ANXA2), Y-box binding protein-1 (YB-1), and nuclear factor erythroid 2-related factor 2 (NRF2), were reported in infertile men with unilateral varicocele [82]. These proteins are involved in processes relevant to sperm function and OS; however, the reported exosomal alterations remain largely correlative, have not been independently validated, and their functional significance in varicocele has not been established.

Overall, current evidence for exosome involvement in varicocele is preliminary and insufficient to establish a causal role. Further independent studies are required to determine whether the reported exosomal alterations have mechanistic or biomarker relevance.

### 5.3. Emerging Therapeutic Applications in Male Infertility

In addition to being investigated as potential diagnostic biomarkers, exosomes have recently attracted interest as experimental therapeutic approaches for male infertility. Preclinical studies, predominantly using in vitro and animal models, suggest that mesenchymal stem cell-derived exosomes promote the restoration of spermatogenesis following chemically induced testicular injury by enhancing seminiferous tubule regeneration, stimulating germ cell proliferation, and improving fertility outcomes [57]. However, evidence from human studies remains lacking, and the clinical relevance of these findings has not been established.

Exosomes have also demonstrated protective effects during sperm cryopreservation by reducing OS, preserving plasma membrane integrity, minimizing DNA damage, and improving post-thaw sperm motility and fertilization capacity in experimental studies [82,83]. These findings remain preclinical and require validation in human reproductive settings before their potential clinical utility can be determined.

Recent studies have highlighted the importance of tissue-specific extracellular vesicles (Ti-EVs), which retain molecular characteristics associated with their tissues of origin and provide valuable insights into intercellular communication within the testicular microenvironment. Investigating Ti-EVs under both physiological and pathological conditions may improve our understanding of disease pathogenesis and inform the investigation of potential therapeutic strategies for male infertility [84].

Beyond their potential applications in regenerative medicine, exosomes are being investigated as experimental delivery platforms because of their capacity to transport proteins, nucleic acids, and other bioactive molecules. Engineering stem cell-derived exosomes to modify their cargo or surface properties represents an experimental strategy for improving therapeutic delivery [85,86]. However, efficient and reproducible cargo loading, control of biodistribution, and tissue- or cell-specific targeting remain major unresolved challenges. Moreover, the safety, immunogenicity, manufacturing reproducibility, and therapeutic efficacy of engineered exosomes require further investigation before clinical translation can be considered.

In conclusion, current evidence suggests that exosome-mediated signaling may contribute to spermatogenesis, sperm maturation, and male reproductive function. Alterations in exosomal molecular cargo have been associated with multiple causes of male infertility, while experimental advances in exosome engineering and regenerative medicine warrant further preclinical investigation [2,35,73]. Well-designed studies, including subsequent human investigations, will be required to determine whether these approaches can ultimately provide clinically relevant diagnostic or therapeutic strategies for male infertility.

The main molecular changes in exosomes associated with male reproductive diseases, along with their biological significance and potential medical applications, are provided in Table 3.

## 6. Exosomes in HPV-Related Reproductive Health

Persistence of high-risk HPV infections is one of the key risk factors for the development of cervical cancer and has also been investigated in relation to reproductive health. Besides being a known risk factor for cervical cancer development, persistent HPV infection has been associated in some studies with adverse reproductive outcomes, including reduced fertility, altered implantation, and adverse obstetric outcomes. However, the available epidemiological evidence remains heterogeneous and, in several areas, inconsistent, with limitations including small sample sizes and differences in study populations and designs. Therefore, a causal relationship between HPV infection and impaired fertility has not been established. Growing evidence suggests that exosomes function as key mediators of intercellular communication between HPV-infected cells and the surrounding tissue microenvironment [21,89,90,91].

### 6.1. Exosome-Mediated Host–Virus Interactions

Exosomes have recently emerged as important mediators of host–pathogen interactions because they transport proteins, lipids, mRNAs, miRNAs, lncRNAs, and other regulatory molecules from infected to uninfected recipient cells. During viral infection, exosomes can exert both protective and pathogenic effects depending on their molecular cargo [37,92,93,94]. Exosomes may exert context-dependent effects during viral infection by transferring viral or host-derived molecules that influence viral propagation and immune responses. Depending on their molecular cargo and cellular origin, these effects may favor either viral persistence or antiviral defense through modulation of innate and adaptive immunity and inflammatory signaling [37].

The dual biological role of exosomes has been observed in a variety of viral infections and provides important insights into the mechanisms potentially relevant to HPV pathogenesis. Exosomes serve as intercellular communication vehicles among epithelial, stromal, and immune cells, and may modulate the tissue microenvironment and influence the balance between viral clearance and persistence [37,92].

### 6.2. Exosomal Molecular Cargo in HPV Infection

Recent evidence indicates that exosomes released by HPV-infected and HPV-associated transformed cells may contain viral nucleic acids, oncoproteins, and regulatory non-coding RNAs capable of influencing gene expression and altering the phenotype of recipient cells. By transferring proteins, nucleic acids, and other bioactive molecules, exosomes have been implicated in the regulation of cell proliferation, apoptosis, angiogenesis, epithelial-to-mesenchymal transition (EMT), and inflammatory signaling in recipient cells [21,37]. However, much of the mechanistic evidence derives from HPV-positive transformed or cervical cancer cell models. Therefore, it remains difficult to distinguish exosomal alterations directly induced by HPV infection from those arising secondary to cellular transformation and the associated tumor microenvironment. Findings from HPV-associated cancer models should consequently not be directly extrapolated to uncomplicated HPV infection.

Host- and HPV-derived miRNAs and lncRNAs have been extensively investigated for their potential roles in signaling pathways involved in immune regulation, viral persistence, and cervical carcinogenesis. Exosomal transfer of these regulatory RNAs has been reported to influence both HPV-associated cells and neighboring recipient cells [21,95,96]. Nevertheless, the extent to which these molecular signatures are specific to HPV infection, rather than reflecting transformation-associated cellular changes, requires further investigation.

### 6.3. Immune Modulation and Persistent Infection

A hallmark of persistent HPV infection is the ability of the virus to evade host immune surveillance, thereby establishing chronic infection and promoting disease progression. Exosomes play a central role in this process by mediating intercellular communication between HPV-infected epithelial cells and the local cervical immune microenvironment. This process is mediated through the transfer of immunomodulatory proteins and regulatory RNAs that suppress antigen presentation, cytokine production, NK cell activity, and T-cell activation [37,97].

Similar mechanisms of immune evasion have also been described in other reproductive disorders, particularly endometriosis, where exosomes modulate macrophage and NK cell function, thereby promoting lesion survival and disease progression. This demonstrates that immune evasion is a biological process that is involved in both HPV infection and chronic reproductive diseases [98,99].

### 6.4. Exosomes and HPV-Associated Reproductive Dysfunction

Apart from cervical carcinogenesis, several studies indicate that HPV infection can negatively impact reproductive health. Persistent infections have been linked to poor implantation, diminished fertility, abnormal pregnancy outcomes, and changes in reproductive tract microenvironment. Exosomes may contribute to the pathogenesis of these disorders by regulating endometrial receptivity, modulating inflammatory responses, and facilitating intercellular communication among epithelial, immune, and stromal cells. While the exact pathways involved in these events have not yet been completely elucidated, exosome signaling is now considered to be an important part of HPV-induced reproductive problems [21,100,101,102].

### 6.5. Translational Implications

The ability of exosomes to reflect the molecular profile of their cells of origin has generated considerable interest in HPV-associated exosomal cargo as a source of minimally invasive biomarkers. The detection of HPV-derived nucleic acids, oncogenic proteins, and regulatory non-coding RNAs in circulating and cervicovaginal exosomes may provide valuable biomarkers for early disease detection, prognostic assessment, disease monitoring, and treatment response [21]. Engineered exosomes have proven themselves to be a very promising tool for the delivery of therapeutic agents—nucleic acids, proteins, and small molecules. All of that makes exosomes among the most promising targets for precision medicine approaches in HPV-related reproductive disorders [41,103].

Generally, current research suggests that exosomes act as important molecular mediators between HPV infection, immune responses, reproductive abnormalities, and cervical cancer development. The ability of exosomes to incorporate both viral and host-derived molecular signals provides important insights into HPV pathogenesis and supports the development of novel diagnostic and therapeutic strategies for HPV-associated diseases [21,37,97].

## 7. Translational Potential of Exosomes in Reproductive Medicine

### 7.1. Exosomes as Diagnostic Biomarkers

Exosomes contain a diverse and relatively stable molecular cargo, including proteins, lipids, mRNAs, miRNAs, lncRNAs, circRNAs, and DNA fragments. Their lipid bilayer protects encapsulated molecules from enzymatic degradation, enabling molecular profiling from a variety of biological fluids, including plasma, FF, seminal plasma, endometrial fluid, and cervical secretions [15,71]. Although these characteristics are potentially advantageous for biomarker development, the superiority of exosome-based biomarkers over conventional diagnostic approaches has not been established.

The molecular cargo of exosomes may provide information related to the physiological or pathological state of their cells of origin; however, exosomal composition is dynamic and can be influenced by biological and environmental factors, as well as pre-analytical variables such as sample collection, processing, storage, and EV isolation methods. Distinct exosomal molecular signatures have been identified in women with PCOS, endometriosis, diminished ovarian reserve, and implantation failure, as well as in men with azoospermia, asthenozoospermia, and varicocele [15,30,71,74,75,104]. However, overlap between molecular profiles in healthy and affected populations may limit the disease specificity of individual candidate biomarkers. HPV-associated exosomes have also been reported to contain viral and host-derived molecular components of potential biomarker relevance [21].

The molecular stability of exosomal cargo and the possibility of obtaining EVs from minimally invasive biological samples have stimulated interest in their potential use in liquid biopsy approaches in reproductive medicine [23,36,105]. Despite these promising findings, exosome-based biomarkers have not yet been established for routine clinical use in reproductive medicine. Current evidence is largely derived from exploratory and observational studies, and robust validation in large, independent clinical cohorts is required before their diagnostic utility and clinical applicability can be established. Furthermore, the practical feasibility and cost-effectiveness of exosome-based diagnostics relative to established methods remain insufficiently evaluated. Requirements for specialized isolation, characterization, and molecular analysis, together with the lack of standardized workflows, currently represent additional barriers to routine clinical implementation.

### 7.2. Therapeutic Potential of Exosomes

Beyond their potential diagnostic applications, exosomes are being investigated as experimental therapeutic delivery platforms because of their capacity to transport and protect bioactive molecules [4,83,85,86]. Preclinical studies suggest that exosomes can be engineered or loaded with specific miRNAs and other therapeutic molecules to modulate cellular signaling pathways. However, these approaches remain experimental, and no exosome-based therapy has been approved for the treatment of reproductive disorders.

Engineered exosomes have been investigated for their effects on granulosa cell viability, promoting oocyte maturation, regulating the PI3K/AKT and ERK signaling pathways, and reducing OS in experimental models of PCOS [13,64]. Similarly, exosome-based delivery systems have been investigated for their potential effects on endometrial receptivity and modulating inflammatory responses in endometriosis and other reproductive disorders [4,30]. These findings are derived predominantly from small-scale preclinical studies and require validation in clinically relevant models and human studies.

Exosomes derived from mesenchymal stem cells have also been investigated in preclinical models of male reproductive dysfunction, with reported effects on spermatogenesis, sperm quality after cryopreservation, OS, and fertilization-related parameters [83,106]. Their ability to transport proteins, lipids, and nucleic acids has additionally motivated investigation of their potential as drug delivery vehicles; however, effective and reproducible tissue-specific targeting remains unresolved.

Moreover, they are being explored as potential carriers for antiviral compounds and genetic therapies against HPV infection. However, these approaches remain primarily experimental and have not yet been established for clinical use [37,107,108]. Notably, safety concerns also remain unresolved, including potential immune reactions, off-target biodistribution and biological effects, unintended transfer of bioactive cargo, and possible tumorigenic effects depending on the source and molecular composition of the administered vesicles. Standardized manufacturing, characterization, dosing, and long-term safety assessment will therefore be required before therapeutic translation. More broadly, although preclinical studies provide a rationale for further investigation of exosome-based interventions, robust clinical evidence remains limited, and no exosome-based therapy is currently approved for reproductive disorders. The current evidence supporting the diagnostic and therapeutic potential of exosomes across reproductive disorders, including HPV-associated disease, is summarized in Table 4.

### 7.3. Challenges and Future Directions

Despite encouraging preclinical results, several important challenges continue to limit the widespread clinical application of exosomes in diagnostics and therapy. One of the major challenges is the lack of standardized protocols for the isolation, purification, characterization, and quantification of exosomes, with considerable methodological variability across studies [4]. Exosomes also represent intrinsically heterogeneous populations whose molecular composition and biological activity may vary according to cellular source, physiological state, culture conditions, and isolation procedures. This heterogeneity complicates the establishment of reproducible quality-control criteria, including standardized measures of identity, purity, potency, and biological activity.

Additionally, improvements in quality control measures, standardized manufacturing processes, and validation through multicenter clinical trials are required to facilitate the clinical translation of exosome-based technologies. For therapeutic applications, large-scale production under Good Manufacturing Practice (GMP) conditions represents an additional challenge, requiring scalable and reproducible methods for vesicle production, purification, characterization, storage, and batch-to-batch consistency. In addition, regulatory challenges related to safety, in vivo biodistribution, cargo-loading efficiency, and long-term effects must be addressed before exosome-based therapies can be widely adopted in clinical practice [4,85,86]. The regulatory pathway may also vary according to the source, degree of manipulation, intended use, therapeutic cargo, and formulation of the exosome-based product, potentially resulting in different regulatory classifications across jurisdictions.

Safety considerations are particularly important in reproductive medicine because the biological activity and biodistribution of administered exosomes may extend beyond the intended target tissue. Off-target uptake or unintended transfer of bioactive cargo could potentially affect gametes, gonadal cells, the endometrium, or other reproductive tissues. Consequently, rigorous assessment of biodistribution, dose-dependent effects, reproductive toxicity, and long-term reproductive and developmental safety will be necessary before clinical application.

Despite these limitations, advances in high-throughput sequencing, proteomics, metabolomics, artificial intelligence, and bioinformatics may improve our understanding of exosomal composition and facilitate the identification of reproducible molecular signatures. The application of these technologies alongside exosome engineering may support the future development of precision reproductive medicine, and contribute to more individualized approaches to diagnostics, prognostics, and treatment of infertility, HPV infection, and other reproductive diseases [27,109,110].

## 8. Discussion

This review highlights the involvement of exosomes in the molecular regulation of reproductive biology. Exosome-mediated communication represents one of several mechanisms of intercellular signaling within the reproductive microenvironment, acting alongside hormones, cytokines, growth factors, and direct cell–cell interactions. Exosomes mediate aspects of the physiological processes of reproduction through the selective transfer of various bioactive molecules, including proteins, lipids, mRNAs, microRNAs, lncRNAs, circRNAs, among others [22,27,29].

Current evidence indicates that exosome-mediated communication is involved across diverse conditions affecting reproductive health. Notably, infertility-related disorders and HPV infection represent distinct clinical and pathophysiological entities and should not be considered equivalent disease processes. Nevertheless, several biological mechanisms relevant to exosome signaling overlap across these conditions, including altered intercellular communication, inflammation, immune dysregulation, OS, aberrant cell signaling, and dysregulated non-coding RNA expression. Emerging evidence suggests that exosomes may contribute to these processes by mediating the transport of signaling molecules and regulatory cargo [23,35,55,80]. This mechanistic overlap provides the rationale for examining these clinically distinct conditions within an exosome-centered framework. However, the relative contribution of exosomes compared with other extracellular and intracellular signaling mechanisms remains incompletely defined, and their involvement should not be interpreted as evidence of a dominant or disease-driving role.

The complexity of the biological structure of exosomes is another reason why their use is becoming increasingly relevant from a translational perspective. Their ability to carry and protect diverse molecular cargo has generated interest in their potential use as biomarkers. However, exosomal cargo does not provide a complete representation of the molecular state of the cell of origin, and the superiority of exosome-based biomarkers over conventional approaches has not been established. Their recovery from biological fluids has motivated investigation of exosome-based liquid biopsies for reproductive disorders [23,29,107].

The therapeutic potential of exosomes is also under investigation. Preclinical studies have demonstrated the potential of engineered exosomes to deliver regulatory RNAs, proteins, and therapeutic molecules, thereby restoring dysregulated cellular signaling pathways. However, most of this evidence derives from cell-based and animal studies, and species-specific differences in reproductive physiology may limit extrapolation to humans. Although these applications remain largely preclinical, exosomes are being investigated as potential delivery systems rather than as established alternatives to conventional platforms. Liposomes, synthetic nanoparticles, and viral vectors offer different advantages and limitations in terms of delivery efficiency, manufacturing control, scalability, immunogenicity, and safety, and current evidence does not establish the overall superiority of exosomes. Engineered exosome-based therapeutic approaches are also being investigated for the treatment of HPV-associated diseases, although these applications remain largely at the preclinical stage [21,41,89,103].

However, there are still some issues that should be resolved to facilitate the transition of exosomes into clinical applications. Considerable methodological variability in exosome isolation, purification, characterization, and molecular analysis complicates direct comparisons among published studies. In addition, commonly used isolation approaches, including ultracentrifugation, may yield heterogeneous EV preparations containing other vesicle subtypes and non-vesicular components. Consequently, findings attributed specifically to exosomes should be interpreted cautiously when their endosomal origin and preparation purity have not been rigorously established. Moreover, the biological heterogeneity of exosomes, together with differences in tissue of origin, disease stage, and sample collection and processing methods, complicates the identification of robust and reproducible biomarkers [6,25,34].

Furthermore, important knowledge gaps remain regarding the fundamental biology of exosomes, limiting their translation into routine clinical practice. Although substantial progress has been made in characterizing exosomal cargo, important questions remain regarding the mechanisms governing cargo selection, tissue-specific targeting, cellular uptake, and the downstream biological effects of exosome-mediated signaling. Addressing these questions will advance our understanding of reproductive biology and facilitate the development of more accurate diagnostic biomarkers and targeted therapeutic strategies [24,25,29]. An additional limitation is that a substantial proportion of mechanistic evidence derives from cell lines and animal models. Although these models are valuable for investigating biological mechanisms, differences between experimental systems and human reproductive physiology limit direct extrapolation to clinical reproductive medicine. Greater validation in well-characterized human populations is therefore required.

Important translational barriers also remain. Clinical implementation will require scalable and reproducible GMP-grade production, standardized quality-control criteria, and appropriate assessment of safety, biodistribution, storage, and batch consistency. The cost and practical feasibility of large-scale exosome production and analysis relative to established diagnostic and therapeutic approaches also remain insufficiently defined. Furthermore, regulatory requirements for exosome-based products may vary according to their source, manipulation, cargo, intended use, and jurisdiction. Given these unresolved methodological, manufacturing, regulatory, and clinical-validation challenges, a reliable timeline for routine clinical translation cannot currently be defined.

In summary, current evidence supports exosome-mediated intercellular communication as a biologically relevant mechanism across normal reproductive physiology, infertility-related disorders, and HPV-associated disease, while these conditions remain clinically and pathophysiologically distinct. Exosomes should therefore be considered as one component of the broader signaling networks involved in reproductive health rather than as central regulators. Their consideration within a common exosome-centered framework may help identify shared molecular pathways as well as disease-specific exosomal signatures. The integration of EV research with genomics, transcriptomics, proteomics, and precision medicine may further advance the development of diagnostic and therapeutic strategies in reproductive medicine [27,109,110].

## 9. Conclusions

Recent evidence has identified exosomes as important mediators of intercellular communication in reproductive physiology, contributing to processes, including folliculogenesis, gametogenesis, fertilization, implantation, and early embryonic development. Alterations in exosome biogenesis, molecular cargo, and signaling have also been associated with female and male infertility. In parallel, emerging evidence indicates that exosomes participate in HPV-associated disease through mechanisms involving intercellular communication, immune and inflammatory regulation, viral persistence, and the transfer of viral and host-derived molecules. Although infertility and HPV infection represent distinct clinical entities, these findings highlight overlapping exosome-mediated mechanisms relevant to reproductive health.

Beyond their biological roles, exosomes are being investigated as potential diagnostic biomarkers and therapeutic platforms because their molecular cargo may reflect the physiological or pathological state of their cells of origin and can mediate the transfer of bioactive molecules. However, most proposed applications remain investigational, and methodological heterogeneity, lack of standardized isolation and characterization protocols, and limited clinical validation currently restrict their translation into routine practice.

Collectively, examining infertility and HPV-associated disease within an exosome-centered framework highlights shared molecular processes while preserving their distinct clinical and pathophysiological characteristics. Further studies integrating extracellular vesicle research with genomics, transcriptomics, proteomics, and bioengineering are needed to define the clinical relevance of these mechanisms and determine whether exosome-based approaches can contribute to precision reproductive medicine.

## Figures and Tables

**Table 1:** Physiological roles of exosomes in the female and male reproductive systems.

Reproductive System	Source of Exosomes	Major Molecular Cargo	Principal Biological Functions	Key Signaling Pathways/Mechanisms	References
Female reproductive system	Granulosa cells	miRNAs, mRNAs, proteins, lipids	Reported involvement in folliculogenesis, granulosa cell proliferation, and steroidogenesis	PI3K/AKT, MAPK/ERK	[13,42,44]
Female reproductive system	Granulosa cells and oocytes	GDF9-, BMP15-associated signaling molecules, miRNAs	Proposed contribution to oocyte maturation and oocyte–granulosa cell communication	TGF-β/BMP	[13,42,45]
Female reproductive system	Follicular fluid EVs/exosome-enriched preparations	Metabolic enzymes, regulatory RNAs	Reported involvement in metabolic regulation, glycolysis, and pathways associated with follicular atresia	Glycolysis, apoptosis, cell-cycle regulation	[13,42,45]
Male reproductive system	Epididymosomes	Proteins, lipids, mRNAs, miRNAs, non-coding RNAs	Proposed contribution to post-testicular sperm maturation, membrane remodeling, motility, and fertilizing capacity	Post-transcriptional regulation	[14,46,50]
Male reproductive system	Epididymosomes	Ubiquitin-associated proteins, enzymes	Reported involvement in sperm quality control, capacitation, and the acrosome reaction	Ubiquitin–proteasome pathway	[14,46,50]
Male reproductive system	Prostasomes	Annexins, transport proteins, antioxidants	Reported involvement in membrane stabilization, OS regulation, immune modulation, and sperm motility	Membrane remodeling, OS regulation	[2,47,51]
Male reproductive system	Seminal plasma EVs/exosome-enriched preparations	Regulatory proteins and RNAs	Potential transfer of paternal molecular information and modulation of sperm function	Cell–cell communication	[2,48,53]

**Table 2:** Molecular mechanisms of exosome-mediated signaling in female reproductive disorders.

Disorder	Major Exosomal Cargo	Principal Molecular Pathways/Targets	Biological Effects	Clinical Implications	References
Polycystic ovary syndrome (PCOS)	miRNAs, lncRNAs, circRNAs	PI3K/AKT, ERK1/2, BMPR1A/Smad1/5/8	Granulosa cell apoptosis, impaired steroidogenesis, altered insulin sensitivity, reduced oocyte quality	Potential biomarkers and engineered exosome-based therapeutic targets	[58,62,64]
Endometriosis	miRNAs, proteins, lipids	PI3K/AKT/mTOR, TGF-β/SMAD, MAPK/ERK, NOTCH, NF-κB/JAK/STAT	Angiogenesis, epithelial–mesenchymal transition, macrophage polarization, progesterone resistance, ectopic lesion survival	Candidate biomarkers for diagnosis, implantation potential, and disease monitoring	[55,67,68]
Endometriosis (immune escape)	MICA/B, ULBP1–3, FasL, TRAIL	NKG2D signaling	NK-cell inhibition, apoptosis of activated immune cells, immune evasion	Novel immunotherapeutic targets	[69]
Diminished ovarian reserve (DOR)	FF-derived miRNAs	PTEN/PI3K/AKT	Enhanced granulosa cell survival, oocyte maturation, follicular development	Assessment of ovarian reserve and reproductive competence	[23,71,72]
Impaired implantation / reduced endometrial receptivity	Exosomal miRNAs	PI3K/AKT and implantation-associated pathways	Reduced embryo implantation, impaired endometrial receptivity	Prediction of implantation success and ART outcomes	[13,23,26]

**Table 3:** Exosome-associated molecular alterations and therapeutic applications in male reproductive disorders.

Male Reproductive Disorder/Condition	Major Exosomal Cargo/Molecular Markers	Affected Signaling Pathways/Mechanisms	Biological Consequences	Potential Clinical Applications	References
Azoospermia	Differentially expressed miRNAs	Wnt, MAPK, mTOR	Impaired spermatogenesis, reduced sperm production	Non-invasive diagnosis, prognosis, patient stratification	[76,77,78]
Asthenozoospermia	↓ CRISP, ↑ Glycodelin	Sperm maturation and fertilization pathways	Reduced sperm motility and fertilization capacity	Biomarkers of sperm function	[78,79,80]
Varicocele	↓ KIF5B, ↑ ANXA2, altered androgen receptor, YB-1, NRF2	OS response, extracellular vesicle dynamics	DNA fragmentation, premature acrosome reaction, impaired sperm maturation	Disease monitoring and therapeutic targets	[80,81,87]
Testicular injury	Mesenchymal stem cell-derived exosomes	Regenerative signaling pathways	Recovery of spermatogenesis, seminiferous tubule regeneration	Regenerative therapy	[35,80,88]
Cryopreserved sperm	Stem cell-derived exosomes	Membrane stabilization, OS regulation	Improved post-thaw motility, membrane integrity, fertilization capacity	Assisted reproductive technologies (ART)	[35,82,83]
Tissue-derived extracellular vesicles (Ti-EVs)	Tissue-specific proteins and RNAs	Local intercellular communication	Characterization of testicular pathology	Precision diagnostics and therapeutic target discovery	[80,84]
Engineered exosomes	Therapeutic proteins, miRNAs, nucleic acids	Targeted molecular delivery	Targeted delivery of therapeutic cargo and restoration of reproductive function	Personalized medicine, targeted drug delivery, and regenerative therapies	[40,41,85]

↑ Increase; ↓ decrease.

**Table 4:** Current and emerging clinical applications of exosomes in reproductive medicine.

Potential Application	Biological Fluid/Exosome Source	Representative Molecular Cargo	Target Reproductive Condition	Potential Clinical Utility	Current Stage of Evidence	References
Liquid biopsy	Plasma, serum, reproductive fluids	miRNAs, lncRNAs, proteins	Female and male reproductive disorders	Potential non-invasive diagnosis and disease monitoring	Investigational/exploratory clinical evidence	[4,22,51,109]
Ovarian reserve assessment	FF	miRNAs	Diminished ovarian reserve (DOR)	Potential assessment of ovarian function	Exploratory human evidence	[23,71]
Endometrial receptivity assessment	Endometrial/reproductive tract fluids	miRNAs, proteins	Implantation failure	Potential assessment of endometrial receptivity and implantation potential	Investigational/exploratory	[22,105]
PCOS biomarker assessment	FF	miRNAs, ncRNAs	PCOS	Potential molecular characterization and biomarker development	Exploratory/preclinical evidence	[7,55,58,61,62]
Male infertility biomarker assessment	Seminal plasma	miRNAs, proteins including glycodelin	Azoospermia, asthenozoospermia, varicocele	Potential characterization of infertility subtype and sperm-related parameters	Exploratory human evidence	[76,77,79,104]
HPV-associated disease biomarkers	Cervical secretions, plasma	Viral and host-derived miRNAs, proteins, nucleic acids	HPV-associated cervical disease	Potential disease detection, monitoring, and prognostic assessment	Preclinical/exploratory clinical evidence	[20,21,89]
Engineered exosome-based therapy	Bioengineered exosomes	Therapeutic RNAs, proteins, nucleic acids	Reproductive disorders and HPV-associated disease	Potential targeted molecular delivery	Preclinical/experimental	[21,28,36,41]
Regenerative therapy	Mesenchymal stem cell-derived exosomes	Bioactive molecular cargo	Testicular injury / impaired spermatogenesis	Potential restoration of spermatogenesis and testicular function	Preclinical (in vivo/in vitro)	[30,106]
Precision reproductive medicine	Multiple biological fluids	Multi-omics exosomal cargo	Reproductive disorders	Potential biomarker-guided patient characterization	Emerging concept	[22]

## Data Availability

No new data were created or analyzed in this study. Data sharing is not applicable to this article.
